# Development of the adult neurogenic niche in the hippocampus of mice

**DOI:** 10.3389/fnana.2015.00053

**Published:** 2015-05-07

**Authors:** Zeina Nicola, Klaus Fabel, Gerd Kempermann

**Affiliations:** Genomics of Regeneration, German Center for Neurodegenerative Diseases (DZNE) Dresden, and CRTD DFG Research Center for Regenerative Therapy, Technische Universität DresdenDresden, Germany

**Keywords:** hippocampus, stem cell, adult neurogenesis, mouse models, dentate gyrus, precursor cells, plasticity, subgranular zone

## Abstract

When does adult hippocampal neurogenesis begin? We describe the development of the neurogenic niche in the subgranular zone (SGZ) of the hippocampal dentate gyrus. We did so from the perspective of the situation in the adult. Ontogeny of the dentate gyrus is complex and results in an ectopic neurogenic niche that lifelong generates new granule cells. Neurogenesis during the fetal and early postnatal periods builds the dentate gyrus and gives way to activity-dependent “adult” neurogenesis. We used markers most relevant to adult neurogenesis research to describe this transition: Nestin, Sox2, BLBP, GFAP, Tbr2, Doublecortin (DCX), NeuroD1 and Prox1. We found that massive changes and a local condensation of proliferating precursor cells occurs between postnatal day 7 (P7), near the peak in proliferation, and P14. Before and around P7, the spatial distribution of cells and the co-localization of markers were distinct from the situation in the adult. Unlike the adult SGZ, the marker pair Nestin/Sox2 and the radial glial marker BLBP were not overlapping during embryonic development, presumably indicating different types of radial glia-like cells. Before P7 GFAP-positive cells in the hilus lacked the radial orientation that is characteristic of the adult type-1 cells. DCX, which is concentrated in type-2b and type-3 progenitor cells and early postmitotic neurons in the adult, showed diffuse expression before P7. Intermediate progenitor cell marker Tbr2 became restricted to the SGZ but was found in the granule cell layer (GCL) and hilus before. Lineage markers NeuroD1 and Prox1 confirmed this pattern. We conclude that the neurogenic niche of adult neurogenesis is in place well before true adulthood. This might indicate that consistent with the hypothesized function of adult neurogenesis in activity-dependent plasticity, the early transition from postnatal neurogenesis to adult neurogenesis coincides with the time, when the young mice start to become active themselves.

Adult hippocampal neurogenesis attracts considerable attention from neuroscientists and the general public because of its suggestive appeal and presumed relevance for cognition in health and disease. Adult hippocampal neurogenesis occurs only in the subgranular zone (SGZ) of the dentate gyrus of most mammals, whose development is set apart from other hippocampal subregions by several features, including the fact that by virtue of adult neurogenesis, dentate gyrus development in some sense “never ends”. Although similar structures exist in other species, the mammalian dentate gyrus is unique in its connectivity and involvement of adult neurogenesis as a means of plastic adaptability. It seems that the dentate gyrus as we see it in modern mammals developed late phylogenetically and develops late ontogenetically. The term “SGZ” for the neurogenic niche was coined by Joseph Altman in 1975 (Altman, 1975) and the SGZ has since been described in great detail (see overview below). How the adult SGZ relates to its predecessors during preceding stages of brain development, however, has only partly been characterized. Most studies have taken a perspective from embryonic and fetal development. We here in part take the opposite view, originating from the situation in the adult. We propose that the activity-dependently regulated neurogenesis that is found in the adult (“secondary neurogenesis”) is a continuation of, but also clearly distinct from, the neurogenesis that provides the structural development of the dentate gyrus itself (“primary neurogenesis”). An analogous distinction with the terms of primary and secondary neurogenesis was first proposed by Cayre (Cayre et al., 2002).

The pioneering hallmark study on dentate gyrus development of rats was published by Altman and Bayer in 1990 (Altman and Bayer, 1990a,b) and Altman updated his view in a book chapter 20 years later (Altman, 2011). Another rat study by Rickman et al. focused on the distribution of radial glia in the developing dentate gyrus but at that time radial glia-like cells had not been recognized as the stem cells that drive development of cortical structures and lifelong fuel adult neurogenesis (Rickmann et al., 1987). The intricate two-stage nature of how the radial glial scaffold of the dentate gyrus influences the formation of the dentate gyrus has been studied in the classical reeler mice, which lack the crucial signaling molecule Reelin (Brunne et al., 2013). The primary radial scaffold leads the precursor cells from the hippocampal hem to the area of the dentate gyrus, the secondary scaffold forms postnatally and partly transforms into the tertiary neurogenic zone of the adult SGZ (Brunne et al., 2010). The nomenclature is somewhat confusing: secondary (or “adult”) neurogenesis thus originates from the tertiary matrix, whereas the primary and secondary matrices generate primary (i.e., embryonic and fetal) hippocampal neurogenesis.

In their ground-breaking studies, Pleasure and colleagues delineated the development of the murine dentate gyrus on the basis of the expressions of transcription factors and identified a transitional germinative matrix below the pial surface (Pleasure et al., 2000; Li and Pleasure, 2007; Li et al., 2009, 2013). In addition, there are studies that have focused on the effect of single genetic factors that affect the development of the dentate gyrus, most importantly Wnt signaling through LEF1 (Galceran et al., 2000; Li and Pleasure, 2005) or SDF-1 and its receptor CXCR4; the latter, importantly, also during the restructuring of the dentate gyrus after birth that is also of interest in our study here (Lu et al., 2002; Berger et al., 2007).

Hevner and colleagues defined the classical sequence of transcription factors for cortical development, most importantly highlighting the role of Eomes (more commonly referred to as Tbr2) as marker of intermediate progenitor cells in cortical development (Englund et al., 2005; Hevner, 2006), and also applied this to the developing (Hodge et al., 2013) and adult hippocampus (Hodge et al., 2008, 2012).

Our goal here was slightly different from these previous studies. What we would like to present is a synthetic view on the development of marker expression patterns in the dentate gyrus, seen from the particular perspective of adult neurogenesis. When does, as far as indicated by marker expression and morphology, “adult” neurogenesis actually begin? The available, more or less anecdotic evidence seemed to suggest that structurally, adult neurogenesis is in place well before proper adulthood (usually defined by sexual maturity, but see Refs. (Rakic, 2002; Lindsey and Tropepe, 2006) for a more detailed discussion of this problem), but no descriptive confirmation of this idea has been available.

Our intention in the present work was thus to map marker expression in the developing and postnatal dentate gyrus using markers that are in common use for studying adult hippocampal neurogenesis. In particular we included (i) “stem cell markers” Sox2 and nestin; (ii) radial glial markers BLBP and GFAP; (iii) intermediate progenitor cell markers Tbr2 and the most commonly used proxy marker for neurogenesis, Doublecortin; and (iv) the pro-neurogenic transcription factors NeuroD1 and Prox1. Of these, Prox1, which is downstream of Wnt/LEF1 deserved particular attention, because in the adult CNS, Prox1 expression is limited to hippocampal granule cells and involved in controlling the initial differentiation of granule cells (Karalay et al., 2011). Finally, on the stem cell side the link between stem cell properties and radial glial morphology and marker expression deserved additional analysis.

Our aim was to focus on the peri- and postnatal aspects of the development of the dentate gyrus that lead to the formation of the neurogenic niche in the adult SGZ. Towards this aim we investigated two embryonic stages (E16.5 and E18.5), four peri- and early-postnatal time points (P0, P3, P7, and P14) and one time-point in the adult (P30). In addition, for the initial description of adult hippocampal neurogenesis, samples from P60 were used. Our study is descriptive and qualitative, literally aiming at providing a detailed picture of development and adhering to the belief that thorough observation stands at the beginning of detailed further analysis.

## Results

### Key Markers of Adult Hippocampal Neurogenesis

**Figure 1:**
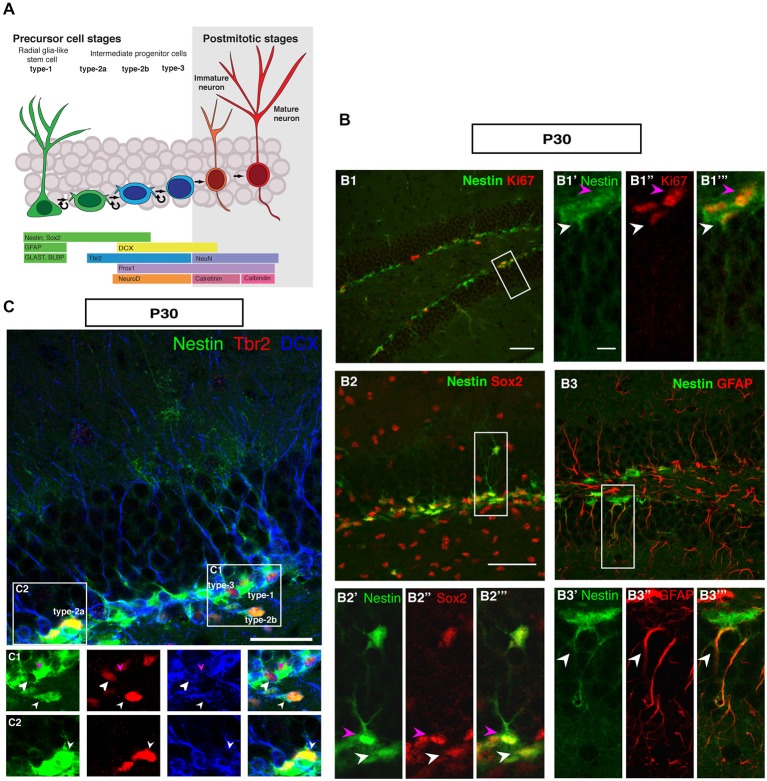
**Cell types in neuronal development of the adult dentate gyrus. (A)** Schematic diagram of neural progenitor cell development in adult hippocampal neurogenesis **(B)** Characterization of type-1 and type-2 cells using proliferation and precursor markers in the adult dentate gyrus. At P30 **(B1–B1‴)** Nestin-GFP (green), Ki67 (red), both type-1 cells with their radial morphology and type-2 cells, which lack the radial process are positive for Ki67 (white and magenta arrowheads respectively). **(B2–B2‴)** Sox2 expression (red) is found in type-1 cells (magenta arrowhead) and in type-2 cells in the SUBGRANULAR ZONE (SGZ) (white arrowhead). **(B3–B3‴)** GFAP-positive cells (red) co-label with Nestin-GFP in type-1 cells in the SGZ (arrowheads). Scale bar, 100 μm for the overview and 20 μm for the insets. **(C)** The co-localization of Nestin-GFP, Tbr2 (red) and DCX (blue) identifies different types of cells in the SGZ of the adult dentate gyrus. **(C1)** Radial glia-like type-1 cells are Nestin-GFP positive but negative for Tbr2 and DCX (arrowhead; magenta), type-2b cells are Nestin-GFP, Tbr2 and DCX positive (arrowhead; small white). The cells, which express only Tbr2 and DCX are type-3 cells (arrowhead; big white). **(C2)** Type-2a cells express Nestin-GFP and Tbr2 but do not express DCX (arrowhead; white). Scale bars are 100 μm for overview and insets.

Figure 1 summarizes the distribution of key markers that characterize adult hippocampal neurogenesis to the level of detail that is considered appropriate to most current studies. The schematic marker progression is found in Figure 1A. The marker panel allows the distinction of rarely dividing or quiescent radial glia-like type-1 cells (Nestin+, Sox2+, GFAP+, Ki67+; Figure 1B), non-radial intermediate or transient amplifying progenitor cells, type-2a (Nestin+, Sox2+), neuronally determined intermediate or transient amplifying progenitor cells, type-2b (Nestin+, Prox1+, NeuroD1+, DCX+), and the less proliferative, optionally migratory neuroblast-like type-3 cells (Nestin-, Prox1+, NeuroD1+, DCX+). The most important more recent addition to that pattern has been marker Tbr2 (Eomes), which labels intermediate progenitor cells (Figures 1A,C; Hodge et al., 2008). Tbr2 is a marker of type-2 cells, also found in few type-1 cells (Hodge et al., 2008).

In the representations in Figure 1, a number of important features of adult hippocampal neurogenesis in mice can be identified. First, the neurogenic niche of the adult SGZ is a very narrow band of cells, only one to three nuclei wide. Second, the SGZ contains the cell bodies of radial elements, whose processes extend through the granule cell layer (GCL) and into the molecular layer (ML). The GCL is densely packed and homogenous. The hilus and the ML are sparsely populated by cells and have sharp boarders to the GCL. The novelty of this panel solely lies in the fact that it brings together the array of common markers side by side. Even at superficial inspection it is obvious that the appearance of the SGZ and the dentate gyrus at this stage is different from earlier stages of development, where the gross appearance is more diffuse and no SGZ is readily discernible.

### Development of the Dentate Gyrus as Visualized with Nestin-GFP Mice

**Figure 2:**
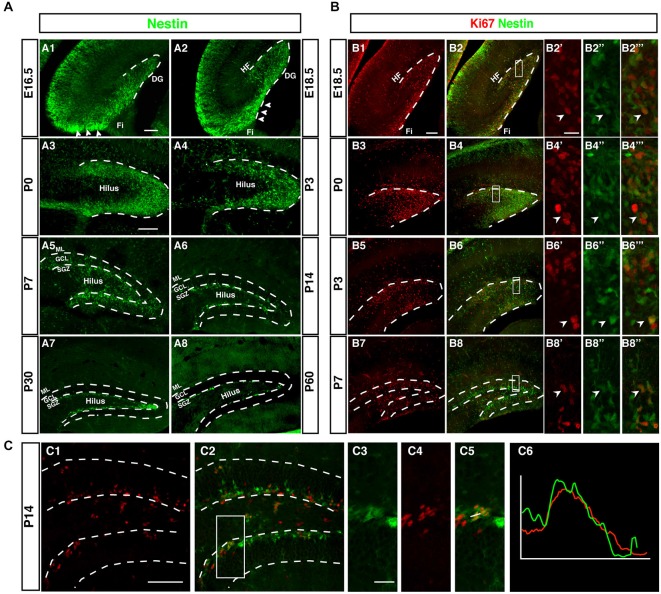
**Development of the dentate neurogenic niche in Nestin-GFP transgenic mice. (A1)** At E16.5 Nestin-GFP positive cells (green) were more concentrated in the area near the fimbria (Fi; arrowheads) and distributed across the hilus. **(A2)** By 18.5, a large number of Nestin- GFP positive cells were found in the area above the fimbria (arrowheads) and extending into the DG. **(A3)** At P0 most of the Nestin positive cells were located on the side of the DG and a few of these positive cells in the hilus. **(A4)** By P3 most of the Nestin-GFP positive cells were distributed along the inner side of the DG. **(A5)** At P7, the SGZ was established and most of the Nestin-GFP positive cells were located in this area, few Nestin-GFP positive cells were found in the granule cell layer (GCL) and hilus. **(A6)** By P14 Nestin-GFP positive cells were restricted to the SGZ. **(A7,A8)** Adult ages (P30, P60), Nestin-GFP positive cells were located only in the SGZ of the DG. **(B)** Expression of the proliferation marker Ki67. **(B1–B2‴)** At E18.5, Ki67 positive cells (red) were found near the fimbria and co-labeled with Nestin- GFP. **(B3–B6‴)** By P0 and P3, Ki67 positive cells were detected in the DG area, and co-labeled with Nestin-GFP **(B7–B8‴)** At P7, Ki67 positive cells were found in the hilus, SGZ and GCL. **(C1–C6)** At P14, the majority of proliferating cells were located in the SGZ and few in the hilus. Ki67 expressing cells in the SGZ co-labeled with Nestin-GFP **(C3–C5)**, as confirmed by the intensity plot of CY3 and GFP **(C6)**. Scale bars are 100 μm for the overview and 20 μm for the insets.

Although Nestin is neither a true “stem cell marker” nor specific to neurogenesis, within the known sites of neurogenesis Nestin is a useful marker for the initial stages of neuronal development. This can be seen in Figure 2A, which outlines the development of the dentate gyrus in Nestin-GFP reporter mice. The pattern based on Nestin-GFP is consistent with the development described by Altman and Bayer in 1990 based on histological stainings and 3H thymidine data (Altman and Bayer, 1990a).

Whereas the remainder of the hippocampus follows a development similar to other cortical structures, as indicated by the layered arrangement of Nestin-positive cells parallel to the ventricular surface, the development of the dentate gyrus begins with a dense cluster of Nestin-GFP expressing cells at the hippocampal hem next to the transition to the future fimbria (Figure 2A1). This primary matrix increasingly spreads out across the extension of the fimbria and towards the pial surface, where a transient matrix forms (Figure 2A2). This is in some continuation with the increasing density of Nestin-GFP expression at the site of the future dentate gyrus, where the bulk of migrating cells bends inwards and dorsally, resulting in the formation of the dorsal blade of the dentate gyrus below the hippocampal fissure (Figures 2A3,A4). Initially, Nestin-GFP-positive cells are at the outside of the future dentate gyrus (being the source of the outside-in pattern of granule cell development at this stage). The putative precursor cells increasingly populate the hilus, which corresponds to the secondary granule cell migration (Figure 2A5). After P0 the migration of Nestin-GFP-positive cells into the hilus dries up. At P7 the demarcation of the developing tertiary matrix (the future SGZ) has begun (Figures 2A6,A7). At P60 Nestin-GFP is restricted to the SGZ and only single cells elsewhere (Figure 2A8). We have previously characterized a population of astrocytes among these Nestin-GFP-positive cells in the ML and in CA1 (Kronenberg et al., 2007); they are unrelated to adult hippocampal neurogenesis.

### Proliferation During Pre-Adult Stages is Diffuse but Spatially Focused in Adulthood

As, by definition, precursor cells have to divide to generate differentiated neurons, cell cycle markers (here Ki67) can be used as a sensitive indicators of the extent of the germinative matrices Figure 2B. In line with other reports and textbook knowledge, proliferation in the ventricular wall is massive during the early embryonic stages. Consistent with the studies by Altman/Bayer and Pleasure/Liu for the site of the dentate gyrus we found this focus of proliferative activity to move towards the pial surface in the E18.5 sample (Figure 2B1). Proliferating cells are now more spread out along the route of the granule cell migration, covering the entire thickness of the future GCL. At P0 the secondary germinative matrix starts forming in the future hilus (Figure 2B3), which in the following 2 weeks becomes wider and increasingly diffuse (Figures 2B5–B8). Nevertheless, at P14 proliferation is almost completely limited to the future SGZ and the hilus, whereas the GCL proper is almost devoid of dividing cells (Figure 2C). As we will see, this pattern is in some contrast to the distribution of putative precursor cell markers. In the adult (compare Figure 1) proliferation is restricted to the SGZ, with the exception of a low number of diffusely distributed dividing cells (mostly astrocytes and NG2 cells; Steiner et al., 2004) that is slightly accentuated in the hilus.

### The SGZ Forms Around P7 and is Clearly Delineated by P14

Thus, both Nestin-GFP and Ki67 data draw a consistent picture that confirms prior knowledge on the subject obtained with other methods (and partly in rats not in mice). What had not been emphasized in the previous studies, however, is the transition between pre- and postnatal hippocampal neurogenesis and neurogenesis in the adult SGZ. In our next step we thus focused on determining at what stage according to the standard definition the SGZ would become visible as a two- to three-nuclei-wide zone below the band of granule cells. Both the data based on Nestin-GFP expression and the detection of proliferative cells indicate that the SGZ starts forming around P7 and is clearly delineated from around P14 onwards (Figure 2C). At this time, Nestin expression and proliferation have almost disappeared outside the SGZ. This might be important because it indicates that the transition between constituting and activity-dependent neurogenesis is relatively sharp.

### Adult Markers are also Found in Pre-Adult Neurogenesis

**Figure 3:**
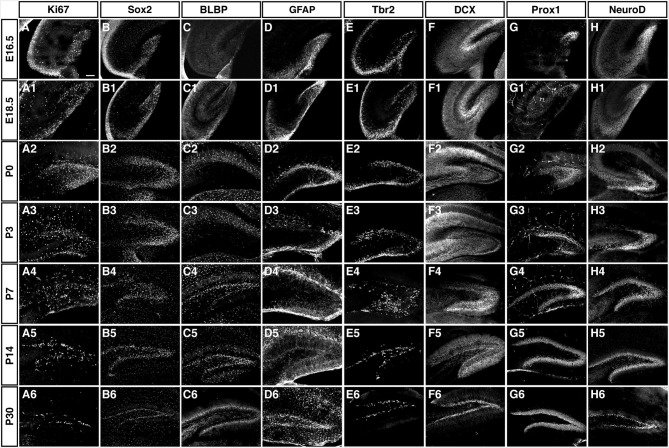
**Overview of the expression patterns of the different precursor, proliferation, intermediate and neuronal markers during DG development**. Ages and markers as labeled in the figure. Scale bar is 100 μm in A.

As depicted in the overview of Figure 3 and the individual characterizations in the remaining Figures, the described markers that are used to characterize adult hippocampal neurogenesis are also found during preceding stages of development. Their individual patterns are described in the following paragraphs. In contrast to the spatial distribution of labeled cells, the marker combinations as such are relatively consistent, but characteristic differences between the stages of primary and secondary neurogenesis emerge.

### BLBP and GFAP/Nestin have a Complementary Expression Pattern Before P7

**Figure 4:**
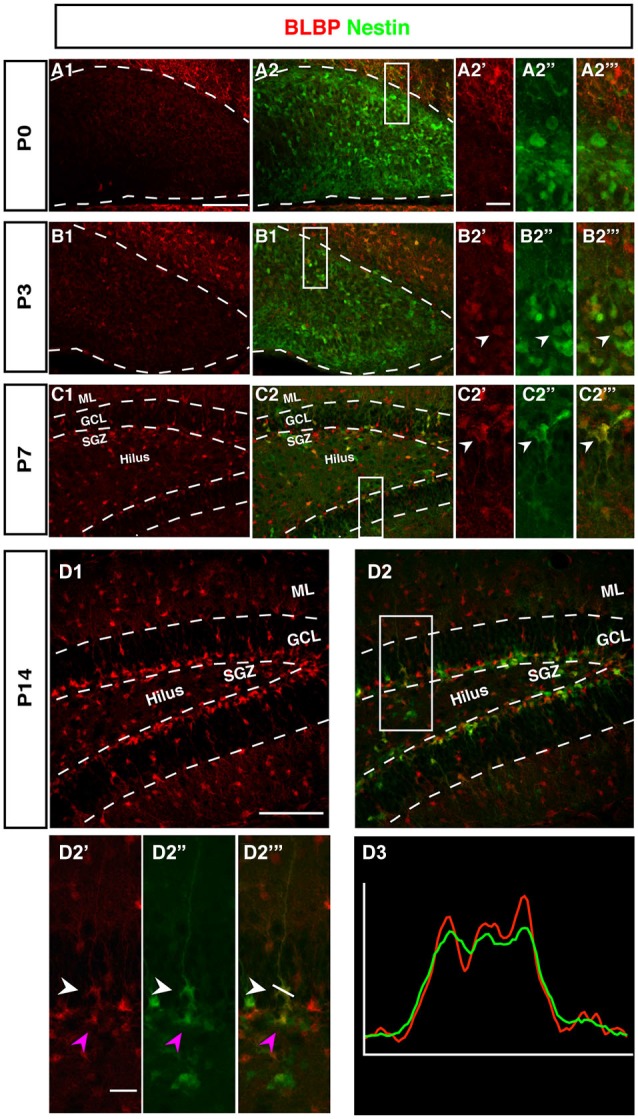
**BLBP expression in the DG during postnatal development of Nestin-GFP mice. (A1–A2‴)** At P0, the expression of BLBP (red) was almost not detected in the DG but Nestin-GFP expression (green) was present. **(B1–B2‴)** At P3, few of BLBP positive cells were found in the DG and some co-express Nestin-GFP (**B2′-B2‴**; arrowhead). **(C1–C2‴)** At P7, BLBP-positive cells were located in the hilus and SGZ of the DG and overlap with some of Nestin-GFP positive cells (**C2′–C2‴**; arrowhead). **(D1–D2‴)** By P14, most BLBP-positive cells were located in the SGZ and co-expressed Nestin-GFP. Radial glia-marker BLBP identifies both type-1 cells with their radial morphology and type-2 cells, which lack the radial processes (**D2′–D‴**; white and magenta arrowheads respectively) the co-labeling was confirmed by comparing the intensity plots of CY3 and GFP **(D3)**. Scale bars are 100 μm for the overview and 20 μm for the insets.

**Figure 5:**
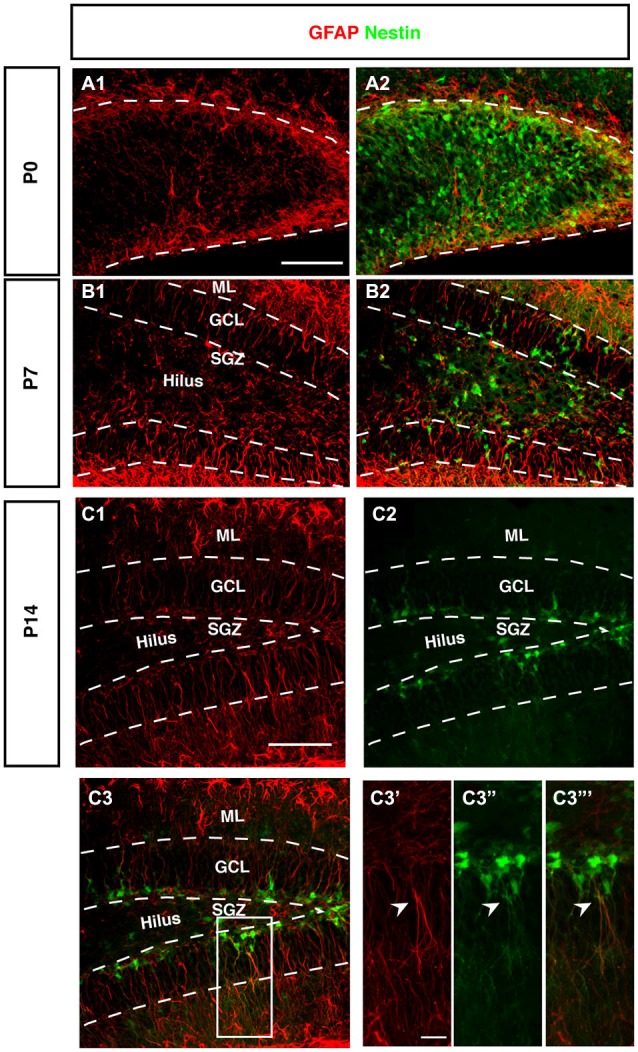
**Expression of GFAP during DG development of Nestin-GFP mice. (A–A2)** At P0, GFAP positive cells (red) were located in the DG area, these positive cells have short processes. **(B–B2)** By P7, GFAP positive cells are found in the hilus and SGZ of the DG. In the SGZ, GFAP-positive cells show long processes extending to the outer GCL and inner molecular layer (ML) and some co-express Nestin-GFP. **(C1–C3‴)** At P14, most of the GFAP-positive cells with radial glial morphology are located in SGZ and co-express Nestin-GFP in type-1 cells (**C3′–C3‴**; arrowhead). Scale bars are 100 μm for the overview and 20 μm for the insets.

The radial marker BLBP showed a particular expression pattern. In adult neurogenesis BLBP co-localizes with GFAP in the radial glia-like type 1 cells and thus co-localizes with both Nestin, GFAP, and Ki67 (see also for example (Steiner et al., 2004; Brunne et al., 2010; DeCarolis et al., 2013). In contrast, here the overlap with Nestin-GFP was essentially absent before P7 (Figure 5). As can be seen in Figure 4, there is a weak overlap at P3, presumably indicating the early stages of the transition to the picture seen at P7 and beyond. Within the region of the future GCL, the first clear signs of BLBP expression in the putative precursor cells were seen at P7 (Figure 4).

Intriguingly, at P0 BLBP labeled numerous cells outside the site of the future GCL and lacking an overlap with Nestin, a pattern that is not seen at later stages. At P0 the margin of the ML towards the future GCL and hilus (where the Nestin-positive cells are seen) is devoid of both Nestin- and BLBP-positive cells. This appears to create a temporal gap in radial glia-like cells at the position where the GCL and the SGZ will form (highlighted in Figures 4B,C). The result is a peculiar separation of the two sets of markers that later merge in the SGZ of the adult.

### Radial Orientation of GFAP-Positive Cells Develops Postnatally

The stem cells of the SGZ are usually named “radial glia-like” in order to emphasize that presumably they are not identical in nature to the classical radial glia of the developing neocortex. The most obvious difference is that the radial glia-like cells of the adult dentate gyrus do not span the entire thickness between the ventricular wall and pial surface. During development this is still the case as long as the primary and secondary matrices are still active (Rickmann et al., 1987). GFAP as canonical astrocyte marker, has been unequivocally related to the precursor cells of the SGZ, resulting in their designation as “astrocyte-like.” Interestingly, at P0 we found that GFAP-positive cells start accumulating in the hilus and future SGZ without showing a clear radial orientation at this time (Figure 5A). This orientation becomes visible at P7 (Figure 5B) and is fully present at P14 (Figure 5C). The developing dentate gyrus is thus transiently devoid of the type of radial elements that are characteristic of the adult situation and fetal cortical development.

### DCX is Diffusely Expressed During Fetal Neurogenesis

**Figure 6:**
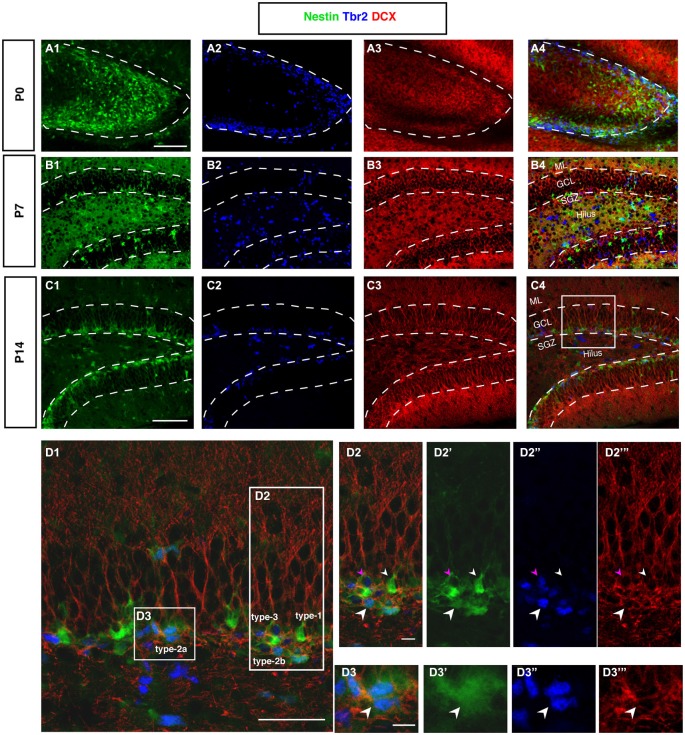
**DCX and Tbr2 expression allows the identification of different stages of neurogenesis during DG development (Nestin-GFP mice). (A–A4)** At P0, Tbr2-positive cells (blue) covered the inner rim of the DG and few of these cells co-labeled with Nestin- GFP (green; A4). In contrast, the expression of DCX (red) was found more in the hilus and DCX-positive cells did not have any processes. **(B–B4)** By P7, most Tbr2-positive cells were found in the hilus and few in the GCL and SGZ and some of these cells co-expressed Nestin-GFP. DCX-positive cells were found in the GCL and showed short processes. **(C–C4)** At P14, the majority of Tbr2-positive cells were restricted to the SGZ and only small numbers were detected in the hilus. DCX-positive cells were found in the SGZ and in GCL but not in the hilus, and these cells had long processes that extended into the ML of the DG. **(D–D3‴)** The co-localization of Nestin-GFP, Tbr2 and DCX identifies different types of cells in the subgraunular zone at P14. **(D2–D2‴)** Radial glia-like type-1 cells are Nestin-GFP -positive but negative for Tbr2 and DCX (arrowhead; small white), type-2b cells are Nestin-GFP, Tbr2 and DCX positive (arrowhead; big white). The cells that express only Tbr2 and DCX are type-3 cells (arrowhead; magenta). **(D3–D3‴)** Type-2a cells express Nestin-GFP and Tbr2 but do not express DCX (arrowhead; white). Scale bars are 100 μm for the overview and 20 μm for the insets.

A very obvious difference between adult and pre-adult neurogenesis is the distribution of DCX. Whereas there is a relatively strong perinuclear expression in the adult and presence of the protein far into the neurites, the distribution is diffuse before P7. Expression within individual cells appears weaker and does not allow the identification of particular cell types. However, it is far more widespread (Figure 6).

### Tbr2-Positive Progenitor Cells in the Hilus and Granule Cell Layer Proper Disappear by P14

In cortical neurogenesis Tbr2 expression marks the population of basal progenitor cells, an intermediate precursor cell type, at least partly capable of a terminal neurogenic division. In adult hippocampal neurogenesis Tbr2 is expressed in type-2 progenitor cells and downregulated thereafter (Hodge et al., 2008). Before P7, Tbr2 is found in a large number of cells throughout the entire area of the developing dentate gyrus (Figure 6A2). Tbr2 marks the route of granule cell migration, indicating that the majority of migrating cells are advanced progenitor cells and (consistent with the above-mentioned results) not glia-like. Tbr2-positive cells are diffusely distributed at this stage (Figures 6A2,B2). They later become increasingly focused in the developing SGZ of P14 onwards (Figure 6C). Figure 6D displays the expression of Tbr2 in the different progenitor cell types found in adult neurogenesis.

On a side note, whereas cell proliferation and nestin-expression remained detectable in the hilus after P14, this was not the case for Tbr2 (not shown). This is in line with the idea that the remaining other nestin-expressing precursor cells are NG2 cells and form a separate lineage. As observed for the other markers, a condensation to the SGZ occurs, further indicating the increasing restriction of neurogenic permissiveness to the SGZ around P7/P14.

#### Prox1 is Expressed in Lineage-Determined Precursor Cells in All Granule Cells

**Figure 7:**
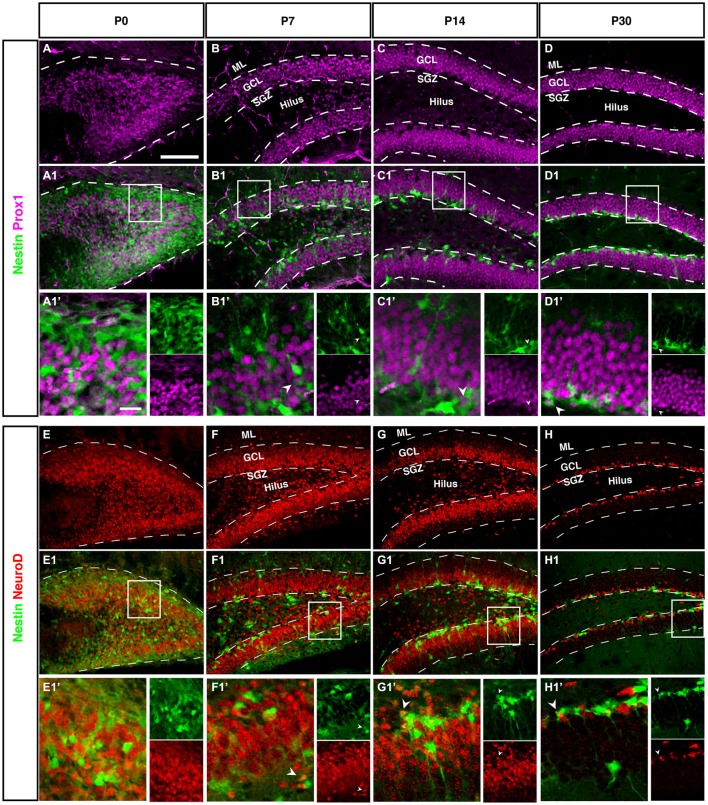
**Prox1 and NeuroD expression in the dentate gyrus during postnatal development of Nestin-GFP mice. (A–D1′) Prox1 expression: (A–A1′)** At P0, Prox1 expression was detected in the DG area and it was more in the hilus than in the side of the DG. Some Prox1- positive cells expressed Nestin-GFP in the DG. **(B–B1′)** By P7, most Prox1-positive cells were located in the GCL and a few in the hilus, whereas most Nestin-GFP-positive cells were found in the SGZ. Few Prox1-positive cells co-labeled with Nestin-GFP (type-2; **B1′**; arrowhead).**(C–C1′)** At P14, Prox1-positive cells were more often found in the GCL than in the hilus, and Nestin-GFP-positive cells were restricted to the SGZ. Some Prox1-positive cells co-expressed Nestin-GFP (type-2; **C1′**; arrowhead). **(D–D1′)** At P30, all Prox1-positive cells were restricted to the GCL whereas Nestin-positive cells were restricted to the SGZ. Also, at this age, double labeling between Prox1 and Nestin-GFP in some of type-2 cells (**D1′**; arrowhead) was detected. **(E–H1′)** NeuroD expression: **(E–E1′)** At P0, NeuroD-positive cells covered the DG area and some of these positive cells showed an overlap with Nestin-GFP expression. **(F–F1′)** At P7, most NeuroD-positive cells were located in the SGZ, GCL and a few in the hilus, whereas Nestin-GFP positive cells were found more in the SGZ than the hilus and GCL. Some NeuroD-positive cells co-expressed Nestin-GFP (type-2; **F1′**; arrowhead). **(G–G1′)** At P14, the majority of NeuroD-positive cells were found in the SGZ, GCL and a few in the hilus, whereas Nestin-GFP-positive cells were located in the SGZ. Some NeuroD-positive cells co-labeled with Nestin-GFP (**G1′**; arrowhead). **(H–H1′)** At P30, all NeuroD and Nestin-GFP-positive cells were restricted to the SGZ. Few NeuroD-positive cells co-expressed Nestin-GFP (type-2; **H1′**; arrowhead). Scale bar is 100 μm.

Prox1, in the adult brain is highly specific for granule cells and the precursor cells from type-2b onwards (Figures 7A–D). Prox1 is required for the maturation of granule cells during development and for maintenance of the intermediate progenitor cells during adult neurogenesis (Lavado et al., 2010; Karalay et al., 2011). We have previously confirmed that in the adult the Prox1-expressing precursor cells acutely respond to extrinsic stimulation (Steiner et al., 2008). Prox1 stays strongly expressed in mature granule cells, and the function of its expression at this stage is not known. At P0 the Prox1 expression pattern highlights the outside-in gradient of neurogenesis, still detectable at this stage. The Prox1-positive Nestin-negative cells are on the outside (Figure 7A).

At P0 we saw a massive accumulation of Prox1-positive cells in the future hilus and first signs of condensation at its outer rim. This pattern is similar to the Tbr2 and NeuroD expression (see next paragraph), highlighting the expression of Prox1 in precursor cells.

#### Lineage Markers NeuroD1 Confirms Prox1 and Tbr2 Findings

Like Tbr2, Neurod1 is a marker for basal progenitor cells, including the cortex. In adult hippocampal neurogenesis, Neurod1 is expressed from the type-2b stage onwards and remains on, albeit at a low level of expression (Figure 7H). Accordingly, we saw the exact same onset as with Tbr2 and a pattern identical to the one of Prox1. This is in line with results from a previous study from our group (Steiner et al., 2006) as well as another more recent reports (Aprea et al., 2014).

## Discussion

The data set presented in this study provides a comprehensive overview of the changing expression of nine marker molecules along the course of the development of the dentate gyrus and at the transition between pre- and early postnatal neurogenesis during which the dentate gyrus is constituted and adult hippocampal neurogenesis emerges. Our key observation is that, structurally, the neurogenic niche of the SGZ forms as early as between P7 and P14, resulting in the fact that “adult” neurogenesis is actually in place well before adulthood has been reached. This is in line but also at some possible discrepancy with results by Gilley et al., who also used Nestin-GFP transgenic mice and both *in vivo* and flow cytometric analysis to characterize precursor cells from P7 and P28 and found that above a potentially stable cell-autonomous baseline there were also changes in transcriptional profiles and precursor cell properties between P7 and P28, suggesting further differentiation (Gilley et al., 2011). Due to the different methodologies used, the detailed relation to our data is difficult to establish at this time. Our results certainly do not rule out that after P14 there are additional relevant changes to the SGZ and its precursor cells. Our point is only that the morphological pattern has emerged by that time.

Speculatively, it might be more than a coincidence that the activity-dependent form of hippocampal neurogenesis, which lasts lifelong, is established at approximately the time, when the newborn rodents have opened their eyes and start to become active. This would suggest that adult neurogenesis is indeed a neurogenesis related to experience and activity in general and not simply linked to adulthood *per se*. The suggestive correlation might hint at an underlying, potentially shared or even direct causality.

Regulation was not the topic of the current study and there are obviously many postnatal changes in connectivity that might contribute to the continued formation of the dentate gyrus and its neurogenic niche. For example, commissural fibers reach the dentate gyrus also around P15 and might have an impact on the transition, and so might have the establishment of input from ipsi- and contralateral mossy cells (Fricke and Cowan, 1977; Ribak et al., 1985). On the other hand, these might depend on the changes in extrinsic input as well. Here our finding is that a characteristic change towards the morphological appearance that remains to be found throughout adulthood, occurs much earlier than commonly assumed.

The term “adult neurogenesis” will stick nevertheless; and rightfully so, given that experience- and activity-dependent neurogenesis is indeed found throughout adulthood. As such it remains an exception from the rule that the adult mammalian brain is largely non-neurogenic.

In this descriptive study we have looked at the development of the dentate gyrus from the perspective of markers that are commonly used to characterize adult hippocampal neurogenesis, highlighting the interesting transition that takes place towards adult neurogenesis.

Besides the obvious differences in gross morphology and distribution of precursor cells, we found a number of more subtle distinctions that set adult neurogenesis apart from the constituting neurogenesis that forms the hippocampus and the dentate gyrus. The most important finding is the temporal heterogeneity of radial glia and radial glia-like cells. We found that only very few of the stem cells expressing markers Sox2 and Nestin showed co-localization with the classical radial marker BLBP before P7 as they do in the adult. Likewise, the orientation of radial elements undergoes a dramatic change. This is in line with a lineage-tracing study, which investigated this transformation from the perspective of gliogenesis in the dentate gyrus (Brunne et al., 2010).

During embryonic and fetal development of the dentate gyrus GFAP-positive cells with long processes populate the entire area. At P7 a hilar population of putative precursor cells has appeared, which later recedes. These GFAP-positive cells lack long processes. A new radial orientation is only established with the formation of the SGZ and the new radial-glia like cells have a distinctive marker pattern besides their particular morphology.

Although well described, the switch from an outside-in to an inside-out gradient of granule cell development is still not well understood. This switch takes place between P0 and P7 (compare Figure 3). Interestingly, at this time, the conditions also have to be created that allow the germinative matrix to concentrate in the SGZ. Consequently, the entire SGZ forms de novo by concentrating several cell types to a narrow band of tissue. It is not clear how this process might be controlled. An important previous study pointed towards the transient but critical role of a more or less diffuse matrix of precursor cells at early postnatal stages (Namba et al., 2005).

A key candidate for regulatory centers involved in this transition are Cajal Retzius cells, which are Reelin secreting pioneer neurons that are also present during the development of the dentate gyrus. In addition, Reelin is expressed by hilar interneurons, including the basket cells (Pesold et al., 1998). Intriguingly, Cajal Retzius cells, like the intermediate precursor cells of adult neurogenesis, express Tbr2. In conditional Tbr2 knockout mice, the transitional subpial matrix did not form and no germinative matrix in the SGZ could form (Hodge et al., 2013). This indicates that factors like Tbr2 play different roles at different stages of neurogenesis in the dentate gyrus. The role of Reelin in this context is underscored by the many findings relating Reelin to radial migration and the proper positioning of granule cells (Zhao and Frotscher, 2010), especially in the context of epileptogenesis (Müller et al., 2009; Duveau et al., 2011). The function of Cajal-Retzius cells, in turn appears to depend on activity in primordial neuronal networks, including GABAergic input onto the Cajal-Retzius neurons (Quattrocolo and Maccaferri, 2013). Reelin is not limited to its function in Cajal-Retzius neurons, but is found more widely in the extracellular matrix of the niche and is also important for neuronal maturation in adult hippocampal neurogenesis (Teixeira et al., 2012).

These latter results clearly indicate that the structural descriptions of development and the mechanistic studies of the underlying molecular mechanisms will have to be complemented by functional studies. In this network sense, the transition from pre-adult to adult neurogenesis remains an almost entirely uncharted terrain. If, however, adult neurogenesis indeed sets in as early as around P15, when the animals start to freely move and explore with open eyes, and if it can be confirmed that even at this early stage, regulation of neurogenesis is activity-dependent (as the available data would suggest) the network situation at the transition time point will also have to be such that it enables the new activity-dependent regulation. Here, again, GABAergic interneurons play a central role in controlling development (Song et al., 2012, 2013).

The aims of the present study were descriptive and our data provide a solid morphological foundation for future studies that are more mechanistically oriented. Our results clearly point to the fact that more research needs to be done on the time-point around P14 and the initiation of a predominantly activity-dependent regulation of neurogenesis. We show that adult neurogenesis is not a simple continuation of embryonic and fetal neurogenesis in the region of the dentate gyrus. A new structure is established which corresponds to a different functionality than before. At the same time, there is also continuity and the adult SGZ is one of the endpoints of the development of the dentate gyrus after E15. This interesting discrepancy must be the topic of future research.

## Materials and Methods

### Animals

For this study we used Nestin-GFP mice, which express the green fluorescent protein (GFP) driven by regulatory elements of the Nestin gene (Yamaguchi et al., 2000). All animals had unlimited access to food and water, and were kept in the same room under a 12 h light/dark cycle. All local and federal regulations regarding animal welfare were followed. The research was approved by the responsible local authority, Regierungspräsidium Dresden. For all experiments, 7 different age groups, ranging from embryonic to adult, were used. At the embryonic stage, there were 2 age groups: E16.5 (4 embryos from the same litters) and E18.5 (4 embryos from the same litters). The postnatal stage contained 5 age groups: P0, P3, P7, (3 mice per age) and P14, P30 (2 mice per age). In addition, one analysis was done at P60 (2 mice). All age groups contained male and female mice.

### Preparation of Brains for Immunohistochemistry

The brains of the mice at ages E16.5, E18.5, P0, P3 and P7 were removed from the skulls, Post-fixed in 4% paraformaldehyde for 24 h at 4°C and then stored in 1XPBS at 4°C. Mice at P14 and P30 (and P60) were deeply anesthetized with ketamine and xylazine and perfused with 0.9% NaCl solution followed by 4% paraformaldehyde in 0.1 M phosphate buffered saline. After that the brains were dissected free from the skull and placed in 4% paraformaldehyde for 24 h and then transferred in 1XPBS and stored at 4°C.

Before sectioning, the brains were embedded in 5% Agar-Agar (in PBS) then glued with Roti Coll 1 to the cutting plate of a semiautomatic vibratome. The cutting plate was fixed into the vibratome chamber, which was filled with 1XPBS and the brains were sliced in the coronal plane into 40 μm thick sections.

### Immunohistochemistry

For immunofluorescence, every 6th section was collected and washed in PBS. The sections were then incubated for 2 h in blocking buffer PBS-plus (10% donkey serum, 0.2% Triton-X 100 in PBS) to block unspecific binding sites and to permeabilize the tissues. After the blocking step, the sections were incubated overnight at 4°C with the primary antibodies diluted in incubation buffer (PBS with 3% donkey serum). The following primary antibodies and concentrations were used: rabbit-anti GFAP (Z0334, 1:1000, Dako), goat-anti Doublecortin (sc-8066, 1:250, Santa Cruz Biotechnology), mouse-anti Prox1 (MAB5654, 1:500, Chemicon International), rabbit-anti Ki67 (NCL-Ki67p 1:500, Novocastra), rabbit-anti Tbr2 (23345, 1:800, Abcam), goat-anti Sox2 (sc-17320, 1:200, Santa Cruz Biotechnology), goat-anti NeuroD (sc-1086, 1:400, Santa Cruz), rabbit-anti BLBP (ab32423, 1:400, Abcam). After several washes in PBS, the sections were incubated for 4 h at room temperature in secondary antibodies diluted in incubation buffer. The concentration of secondary antibodies CY3 (711-495-152, Jackson Immuno Research) and CY5 (715-175-15, Jackson Immuno Research) was 1:500. After the incubation, sections were washed several times in PBS and incubated in DAPI (861405, 1:4000, Invitrogen) for 10 min, then washed again in PBS and mounted on slides in Aqua Poly/Mount.

### Imaging and Image Processing

Images were acquired using an upright Zeiss Axioimager with an ApoTome.2 unit and a Zeiss LSM 780 on an Examiner stand using ZEN black 2012.

The ApoTome images were acquired using a 10x/0.45 and 20x/0.75 objectives.

On the LSM 780, images were acquired using a Plan-Apochromat 20x/0.8 air objective and DAPI, GFP, Cy3 and Cy5 were excited using the laser lines 405 nm, 488 nm, 561 nm and 633 nm, respectively. For emission detection the following wavelength areas were used: DAPI: 415–450 nm, GFP: 499–534 nm, CY3: 588–623 nm and CY5: 649–690 nm.

Images were processed offline using Fiji (National Institute of Health) and Adobe Photoshop CS5®(Adobe Systems Incorporated). The image composites and the figures were assembled using Adobe Illustrator CS4. Figures were not digitally manipulated otherwise.

Co-localization analysis was done using line profiles where the pixel intensity along the line of interest of each channel was determined and plotted using Fiji and Prism software. For this, single optical sections were chosen from the complete z-stack and similar shapes of line profiles were assumed to point towards co-localization.

## Author Contributions

ZN performed the experiments, the imaging and data analysis. KF designed the experiment and performed data interpretation. GK designed the experiment and prepared the manuscript.

## Conflict of Interest Statement

The authors declare that the research was conducted in the absence of any commercial or financial relationships that could be construed as a potential conflict of interest.
